# Evaluation of MCT1, MCT4 and CD147 Genes in Peripheral Blood Cells of Breast Cancer Patients and Their Potential Use as Diagnostic and Prognostic Markers

**DOI:** 10.3390/ijms18040170

**Published:** 2017-03-23

**Authors:** Maria Cláudia de B. Luz, Matheus M. Perez, Ligia A. Azzalis, Luiz Vinícius de A. Sousa, Fernando Adami, Fernando L. A. Fonseca, Beatriz da C. A. Alves

**Affiliations:** 1 Clinical Laboratory, Faculdade de Medicina do ABC (FMABC), Av. Príncipe de Gales, 821, CEP 09060-650 Santo André, SP, Brazil; mb.luz@uol.com.br (M.C.d.B.L.); matheusmpontoperez@gmail.com (M.M.P.); profferfonseca@gmail.com (F.L.A.F.); 2 Biological Science Department, UNIFESP, Rua Prof. Artur Riedel, 275, CEP 09972-270 Diadema, SP, Brazil; laazzalis@uol.com.br; 3 Epidemiology Laboratory and Data Analysis, FMABC, Av. Príncipe de Gales, 821, CEP 09060-650 Santo André, SP, Brazil; luiz.sousa@fmabc.br (L.V.d.A.S.); fernando.adami@uol.com.br (F.A.)

**Keywords:** biomarkers, prognosis, metastasis, breast cancer, monocarboxylate transporter (MCT), hypoxia

## Abstract

Background: Patients with breast cancer—the deadliest cancer among women—are at constant risk of developing metastasis. Oxidative stress and hypoxia are common feature of tumor cells that can proliferate even in a resultant metabolic acidosis. Despite the low extracellular pH, intracellular pH of tumor cells remains relatively normal, or even more alkaline due to the action of a membrane protein family known as monocarboxylate transporters (MCTs). The objective of this study was to verify the diagnostic and prognostic value of *MCT1*, *MCT4* and *CD147* in tumor and peripheral blood samples of patients with breast cancer undergoing chemotherapic treatment. Methods: Differential expression of *MCT1*, *MCT4* and *CD147* obtained by qPCR was determined by 2^−ΔΔ*C*q^ method between biological samples (tumor and serial samples of peripheral) of patients (*n* = 125) and healthy women (*n* = 25). Results: tumor samples with higher histological grades have shown higher expression of these markers; this higher expression was also observed in blood samples obtained at diagnosis of patients when compared to healthy women and in patients with positive progression of the disease (metastasis development). Conclusion: markers studied here could be a promising strategy in routine laboratory evaluations as breast cancer diagnosis and prognosis.

## 1. Introduction

Breast neoplasias are the major cause of cancer mortality among women, accounting for 25% of new cancer cases that arise every year. Tumor cells are able to spread throughout the whole body through invasive mechanisms and metastasis. Invasion is related to the direct penetration of tumor cells into adjacent tissues. Metastasis, in turn, refers to the ability of cancer cells to penetrate into lymphatic and blood vessels, circulate through the bloodstream, and then expand to other parts of the body where they grow and form colonies of the primary neoplasia [1]. Nearly 10%–15% of patients develop an aggressive disease course with distant metastasis within the first three years after the detection of the primary tumor. However, it is not unusual that this metastasis detection may occur 10 years or later after the initial diagnosis. Thus, it is logical to conclude that patients with breast cancer are at constant risk of developing metastasis. The heterogeneous nature of these metastases makes it difficult to define not only a cure but also the risk factors for their development. Currently, some prognostic markers are already used in clinical practice, and hormone receptors like estrogen and progesterone receptors (ER and PR), human epidermal growth factor receptor 2 (HER2) [2], Ki-67 antigen [3] and carbohydrate 15-3 and carcinoembryonic antigens (CA 15-3 and CEA) [4] are among the most common. Nevertheless, by adding new metastasis markers to this list, prognosis accuracy can be improved.

Tumor cells are extremely glycolytic, produce excess lactate and proliferate in hypoxic conditions. Oxidative stress and hypoxia, which directly cause a decrease in extracellular pH [5], are known markers of tumor aggressiveness [6]. Despite the low extracellular pH, intracellular pH of tumor cells remains relatively normal, or even more alkaline due to the action of a membrane protein family known as monocarboxylate transporters (MCTs) [7]. MCTs participate in the metabolism of all cell types, but under hypoxic or ischemic conditions, tissues become dependent on this pathway (monocarboxylate transport) to obtain energy. However, tissues such as skeletal muscle, red blood cells and many tumor tissue types depend on this pathway to obtain ATP also under normal physiological conditions [8]. This preference of tumor cells for the glycolytic pathway for the production of energy even under normal physiological conditions is known as the Warburg effect. So according to Warburg effect, tumor cells are able to produce an increased amount of lactate that should be exported from the cell in order to maintain cellular pH [8]. This transport of lactate across plasmatic membrane is performed by MCTs.

The role of MCTs in cell homeostasis in many tissues has already been acknowledged and described [9]; the understanding of this role in tumor biology, however, is still incomplete. The association of malignant tumor progression with metabolic adaptations induced by hypoxia has been pointed as a promising target for therapies [10]. The MCTs family (SLC16A) is composed of 14 members [11]; these membrane proteins transport short-chain monocarboxylates (lactate, pyruvate and ketone) across the cell membrane [8]. In MCT proteins, 12 transmembrane domains are found. Among MCTs members, only MCT1-4 transport monocarboxylates couple with a proton across a cell membrane [7]. Both *MCT1* and *MCT4* have been widely studied in cancer cells and are on the list of metabolic targets for anticancer therapies [9]. Taking into consideration the main role of MCTs in the metabolic adaptations of tumor cells, their inhibition would directly impact on cell pH regulation, thus affecting its viability.

*MCT1* is the most widely expressed and is regulated by its association with the glycoprotein *CD147*. The increase in its expression has been revealed in several types of cancer, such as neuroblastomas [12] and different brain tumors [13]. In patients with breast cancer and p53 mutations, the increased expression of *MCT1* is associated with a worse prognosis [14]. In soft tissue sarcomas, *MCT1* was respectively found in 60.5% and 32.6% of cases in the plasma membrane and in the nucleus in studied subjects. The presence of *MCT1* in the nucleus is associated with a better prognosis whereas in the plasma membrane it represents a worse prognosis [15]. On the other hand, *MCT4* is strongly expressed in glycolytic tissues, such as skeletal muscle fibers, astrocytes, leukocytes, chondrocytes, and some mammalian cell lineages [16,17]. *MCT4* has also been found in soft tissue sarcomas, mostly in the plasma membrane, indicating a worse prognosis, and homogeneously distributed throughout the cell [15]. *MCT1* and 4 are regulated by *CD147*, a glycoprotein involved in the traffic and anchorage of membrane proteins, angiogenesis and extracellular matrix modeling [18]. The absence of *CD147* expression leads to the production of *MCT1* and *MCT4*, but they remain in the endoplasmic reticulum and in the Golgi apparatus [8]. Therefore, the presence of *CD147* is required for the translocation of *MCT1* and 4 to the plasma membrane where they act*. CD147* expression, in turn, is positively regulated by stimuli that increase the metabolic activity of the cell and lead to the stimulation of glycolysis and increased expression of glucose transporters [8]. The prognostic value of *CD147* is apparently associated with its co-expression with *MCT1* in breast and gastric cancer cases [19]. The conclusion is that the activity of MCTs is also hindered by the inhibition of *CD147*, making it a potential target for antitumor therapies [20]. Besides being regulated by *CD147*, *MCT 1* and 4 are also involved in the proper expression of *CD147* in the plasma membrane [21]. Thus, the contribution of MCTs to the malignant phenotype would not be limited to lactate transport and pH regulation, but would also include the role of regulators of *CD147* expression. If the hypothesis is confirmed, MCTs should have an indirect participation in tumor growth and angiogenesis as well as in the migration of tumor cells and in the invasion process [21].

One of the possible monitoring strategies of neoplasias is the study of molecular markers in circulating tumor cells (CTCs) [22,23]. In breast cancer patients, the detection of CTCs in the peripheral blood was associated with a reduced life expectancy. In other words, recent molecular studies showed that the expression of multiple markers in CTCs in peripheral blood has potential prognostic and chemosensitive value [24,25,26]. As CTCs are extremely heterogeneous, the analysis of their molecular characterization through the study of different gene expressions is of utmost importance not only to confirm their origin but also to detect phenotypic alterations associated with tumor progression and chemotherapy resistance.

Molecular diagnostics for the detection and characterization of CTCs, easily performed in routine laboratory work, can be robust and specific. Immunohistochemical and molecular techniques, such as the polymerase chain reaction (PCR) and its variations, such as the reverse transcription polymerase chain reaction (RT-PCR) and quantitative polymerase chain reaction (qPCR), are commonly used for the detection of epithelial cells in patients with breast cancer. Novaes et al. [27] showed that the sensitivity of the RT-PCR technique for the detection of cells that express cytokeratin 19 (CK-19) in the peripheral blood mononuclear fraction (PBMN) was 1 in 10^6^. More recent studies that evaluate multiple markers in peripheral blood confirm the expression of certain CTC markers in the blood as prognosis and chemosensitivity markers [24,26].

Solid biopsy (resection of tumor fragments) cannot be frequently performed due to the fact it is an invasive procedure, and in many cases difficult to be carried out owing to the tumor location. Therefore, it is of great clinical importance that less invasive methods to investigate the dynamics of tumors be developed so that these methods can be periodically applied throughout the treatment process. Liquid biopsy [28], on the other hand, has been drawing the attention of the medical community. It consists of genetic material analysis, both DNA and RNA, of circulating tumor cells in the peripheral blood of patients. The analysis of these cellular characteristics, such as the mutation of DNA or gene expression, obtained from peripheral blood samples may be a valuable tool for cancer monitoring during the course of a treatment. This study aimed to evaluate the potential prognostic value of *MCT1*, *MCT4* and *CD147* expressions by qPCR in peripheral blood of patients with breast cancer under chemotherapy treatment.

## 2. Results

Table 1 shows the characterization of patients included in this study.

Clinical stage was determined at diagnosis according to tumor and clinical characteristics of patients and progression is related to clinical outcome characteristics such as appearance or absence of metastasis, disease recurrence, response to treatment, etc.

### 2.1. Expression Characterization of MCT1, MCT4 and CD147 in Tumor Samples

**Figure 1:**
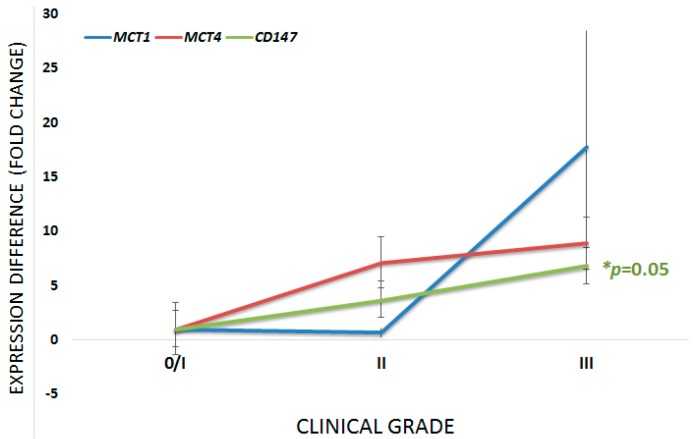
Graphical representation of the difference in expression of the three markers under study according to the clinical stage. For all three cases, the smallest group of stage samples was used as the calibrator. * Kruskal–Wallis test; 95% confidence interval. *p* values are related to the CD147 curve.

First of all, we verified the expression profile in tumor samples and all of which showed expression of the markers here studied. Aiming to associate the expression profile of studied markers with clinical stage, the samples were subdivided into three groups: group stage 0–I (36 samples); group stage II (59 samples); group stage III (30 samples). The differences in gene expression between groups are shown in Table 2 and is graphically represented in Figure 1.

### 2.2. Expression of the Markers in Serial Collection of Peripheral Blood: Potential Use in Diagnosis

In an attempt to evaluate the possibility of using the studied markers in liquid biopsies, the expression profile of these markers in the peripheral blood of healthy women and of patients with cancer (at diagnosis, when samples were collected prior to the beginning of chemotherapy) was performed. The studied markers in the patients’ blood showed increased mean expression in relation to the healthy women. Expression differences, calculated by the 2^−ΔΔ*C*q^ method, can be seen in Table 3.

### 2.3. Profile of MCT1, MCT4 and CD147 Expressions in Patients with Cancer Throughout Treatment: Potential Use as Prognosis Markers

**Figure 2:**
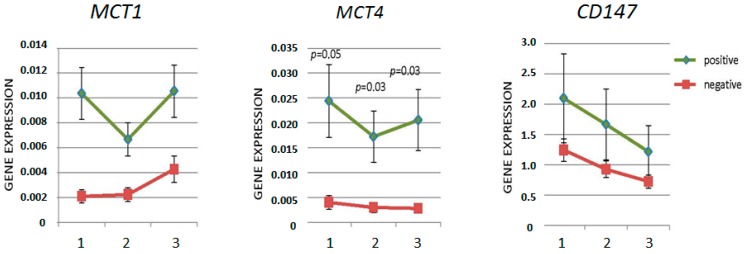
Graphical representation of expression difference between the patients’ blood samples with positive (green; *n* = 10) and negative (red; *n* = 113) progression of the disease. (1) at diagnosis; (2) 3 months after beginning of chemotherapy; and (3) 6 months after beginning of chemotherapy.

For the sake of analyzing the behavior of the markers according to the disease progression, the patients were divided into two groups: one composed of patients with positive progression (disease relapse/metastasis) and the other of patients with negative progression (relapse-free cases). Figure 2 shows the expression of the studied markers in the two groups in the serial collection of peripheral blood. As seen in Figure 2, MCTs and *CD147* markers had increased expression in the peripheral blood samples from patients with positive disease progression when compared to those obtained from patients with negative progression. The greatest difference in expression was observed in the collection at diagnosis, before the beginning of chemotherapy, which suggests that this sample is ideal to evaluate the risk for progression to develop metastasis and disease relapse.

There was no significant relationship between the expression of these markers with other clinical-pathological data than those described here.

### 2.4. MCT1, MCT4 and CD147 Expressions in MCF7 Cells as Laboratory Parameter

The expression of patients and donors was compared to the expression of the markers in MCF7 cells derived from human breast adenocarcinoma. The comparison was made using blood samples at diagnosis, and patients were grouped according to the disease progression *status*. The obtained values are shown in Table 4. The data, when compared to MCF7 cells, show that there is an increase in expression in patients according to the disease progression *status* and that patients have increased expression levels in relation to healthy women. 

## 3. Discussion

The markers here studied are more expressed in tumor of patients with higher clinical grade. These results comply with the ones found by Zhu et al. [29], who associated the expression of *MCT4* with higher clinical grade and worse prognosis in oral squamous cell carcinoma patients. This relation between the increased expression of *MCT1* could also be observed in gastric [19], renal [30] and bladder cancer [31], which once again highlight the role of these markers in the progression of these tumor types. Moreover, they have shown an increased expression in patients’ blood in relation to the blood of healthy women. All genes (*MCT1*, *MCT4* and *CD147*) are expressed by leukocytes under normal physiological conditions, justifying their detection in healthy women. So far, however, studies on the expression of these markers in circulating tumor cells have not been published, and data comparing the expression of these markers in the blood of healthy women cannot be found either. The current study shows that it is possible to detect the expression of these markers in peripheral blood samples of both breast cancer patients at diagnosis and in healthy women. As expected, the studied markers were more strongly expressed in patients with cancer once they are positively modulated by tumor hypoxic conditions. The increase in the expression of these genes in the peripheral blood of the patients may occur due to the presence of circulating tumor cells (CTCs); moreover, our results suggest that *MCT1* and *CD147*—markers that have shown statistical significance—expression in blood samples could be used as a diagnosis marker of breast cancer. The increased expression of these markers in patients with evaluated progression at diagnosis reflects an adaptation of the tumor to the acidosis caused by the activation of the glycolytic pathway and lactate production, and this adaptation prevents the activation of the apoptotic pathway in these patients [32].

To verify if the MCTs and *CD147* could be used in liquid biopsies as prognosis markers, their expression was analyzed in serial samples of peripheral blood obtained from patients undergoing chemotherapy treatment. Patients were split into two groups: one composed of patients with positive progression (disease relapse/metastasis) and the other of patients with negative progression. As shown, *MCT1* is the only marker that has increased expression over chemotherapy (despite a fall after the start of chemotherapy in patients with positive progression, expression is increased during the treatment in both groups of patients evaluated); *CD147*, in turn, had a decreased expression throughout the treatment, demonstrating their susceptibility to chemotherapy. *MCT4* was shown to be a potential candidate for response to the treatment marker, since patients with negative progression showed a steady decline in its expression, while those with positive progression showed an increase of this marker—after falling shortly after the start of treatment—6 months after treatment with expression of difference between the two groups of patients being statistically significant. The fact that *MCT4* expression tended to increase over the treatment of patients with positive progression of the disease may be an indication of non-response to chemotherapy. In fact, resistance to chemotherapy induced by *MCT4* has been demonstrated: in a review, Bovenzi et al., 2015 [33] described 12 studies in which increased expression of *MCT4* is associated with worse prognosis in patients with breast cancer, pancreatic, head and neck, hepatocellular carcinoma, gastric and colon. Drug-resistance mediated by *CD147* was also described *MCT4* by Peng et al., 2015 [34]. Thus, evaluation of expression after six months of chemotherapy can be a supplement for clinical onset of metastasis risk analysis.

Most of the studies that analyze the expression of MCTs and *CD147* do so by using immunohistochemistry on the tumor. Therefore, these studies manage to distinguish whether MCT proteins are in the plasma membrane or in the cytoplasm, retained in the endoplasmic reticulum and in the Golgi apparatus. According to these authors, the presence of *MCT1* in the plasma membrane indicates a worse prognosis. The presence of *MCT4* in the membrane is less frequent than that of *MCT1*, and its role in the cytoplasm has not been totally elucidated yet; however, many authors hypothesize its function in the metabolism of both lactate and pyruvate, another monocarboxylate, in this cellular compartment. Despite the fact that the analysis of expression through real-time PCR (qPCR) does not show the cellular compartment where the proteins are, it predicts disease progression in samples of peripheral blood by means of mRNA expression intensity. Besides, this is a fast, low-cost, very sensitive and specific molecular technique easily performed in clinical laboratories.

Our results show that it is possible to use samples of peripheral blood to assess diagnosis and response to treatment and prognosis since the studied markers are more expressed in patients with disease progression. Indeed, Aaroe et al., 2010 and LaBreche et al., 2011 [35,36] have already shown the expression of peripheral blood cells. Patterns can distinguish breast cancer patients from healthy women All the data presented so far are related to the relative expression analysis between patients and healthy women, between different biological samples of patients or between different time points during the treatment process. For the sake of laboratory analysis, however, there should be another comparison parameter that could be established as standard. Our results suggest that, based on the variation in expression difference levels compared to MCF7, whose cDNA was synthesized from 1.0 μg of total RNA, it can be said that those women with *MCT1*, *MCT4* and *CD147* expressions respectively ranging between 0.01–0.05, 0.0005–0.002 and 0.063–0.072 are considered healthy; those whose *MCT1*, *MCT4* and *CD147* expression levels respectively ranged between 0.08–0.28, 0.001–0.006 and 0.32–0.37 are cancer patients with low odds of progression; finally, samples with results showing relative expression in MCF7 between 0.31–1.09, 0.03–0.08 and 0.53–0.80 respectively for *MCT1*, *MCT4* and *CD147* indicate disease with chances of disease progression. As far as we know, studies that estimate expression level ranges, obtained from the peripheral blood of women, for breast cancer diagnosis and prognosis have not been published yet. While promising, these data must be validated by another cohort of patients and healthy women. If confirmed, these results may be used as complementary laboratory parameters for diagnosis and prognosis.

## 4. Material and Methods

### 4.1. Patients

A total of 125 patients with breast cancer from the Faculdade de Medicina do ABC (FMABC) oncology ambulatory care unit were included (Institutional Ethics Committee protocol number 384/2007; approved on 21 November 2007; informed consent was obtained from all individual participants included in the study; this study has been performed in accordance with the ethical standards as laid down in the 1964 Declaration of Helsinki and its later amendments or comparable ethical standards). Three blood samples were drawn from each patient at diagnosis and time points 3 and 6 months after the beginning of chemotherapy. One peripheral blood sample was obtained from 25 healthy women (controls). A block of paraffin embedded tumor sections (10 μm) was provided by each one of these patients.

### 4.2. Total RNA Extraction from Peripheral Blood

A total of 5 mL of peripheral blood was collected by venipuncture in an EDTA tube. Total RNA was isolated from total blood using TRIzol reagent (TRIzol LS Reagent, Thermo Fisher Scientific cat. no. 10296-010, Waltham, MA, USA) according to manufacturer’s directions. Total RNA concentration was estimated by spectrophotometry (NanoVue Plus—GE Health Care, Buckinghamshire, UK).

### 4.3. RNA Extraction from Paraffin-Embedded Material

Two 10 μm sections per block were obtained with the aid of a microtome from the pathology department of FMABC. For total RNA isolation, RNeasy FFPE kit (Qiagen, cat. no. 74404, Hilden, Germany) was used according to manufacturer’s directions. The extracted material was quantified by a NanoVue Plus spectrophotometer.

### 4.4. cDNA Synthesis

Total RNA samples (1 μg) were converted into cDNA with SuperScript III First Strand qPCR Supermix (Invitrogen, cat. no. 11752050, Carlsbad, CA, USA) following the manufacturer’s protocol.

### 4.5. qPCR

The expression of MCT1 (F-TACCTCCAGACTCTCCTGGC and R-GTCCCCTCCGCAAAGTCTAC), MCT4 (F-CGTTCTGGGATGGGACTGAC and R-ATGTGCCTCTGGACCATGTG) and CD147 (F-CCGTAGAAGACCTTGGCTCC and R-TACTCTCCCCACTGGTCGTC) was evaluated by real-time PCR (qPCR). Specific primers for each selected gene were designed using Primer3 Input 0.4.0 software [37], available at http://frodo.wi.mit.edu/primer3/. For the normalization of the relative expression of target genes, the expression values of housekeeping gene GAPDH (glyceraldehyde 3-phosphate dehydrogenase; F-GACCACAGTCCATGCCAT and R-CAGCTCAGGGATGACCTT) were used. Amplifications was performed in an Applied Biosystems 7500 Real Time PCR Systems thermocycler (Applied Biosystems, Foster City, CA, USA) in a final volume of 15 μL, using 1× of a fluorescent dye (SYBR Green dye-Quantitec SYBR Green PCR kit, Qiagen, Cat No. 204143, Hilden, Germany) 0, 25 μM of each primer, in thermal conditions of an initial *hot start* step at 95 °C for 10 min, followed by 40 cycles of 95 °C for 15 s and 60 °C for 25 s.

### 4.6. Gene Expression Analysis

Gene expressions and differences in the expression in analyzed groups were evaluated by the 2^−ΔΔ*C*q^ method, where *C*q represents the threshold cycle (quantification cycle) [38] and by 2^−Δ*C*q^ [39]. Results are shown as differential expression: a fold change value followed by a range of difference in expression (minimum and maximum).

### 4.7. Statistical Analysis

Qualitative variables were described by absolute and relative frequencies. Due to the non-normal distribution of data (Shapiro–Wilk, *p* < 0.05), medians and the respective confidence intervals of 95% were used to describe the concentration of blood markers. Besides, the Mann–Whitney test was applied in order to analyze concentration differences between groups, the Kruskal–Wallis test was used to analyze the evolution of the concentration of the markers from diagnosis to month 6, and the Spearman correlation test was performed to analyze the correlation between blood markers and the tumor marker at diagnosis. The overall confidence level was of 95%, and the software Stata 11.0 (StataCorp LLC, College Station, TX, USA) was used for statistical analyses.

## 5. Conclusions

Today, the Warburg effect is considered a new characteristic of neoplasias and MCTs play a key role in the glycolytic phenotype and resistance to acidosis in tumor cells. Among the many MCT family members, *MCT1* and *MCT4*, along with the chaperon protein *CD147*, stand out as possible therapeutic targets due to their role in tumor adaptation to the microenvironment. The major aim of this study was to evaluate the behavior of these markers from the peripheral blood samples of cancer patients throughout chemotherapy so that their potential use in liquid biopsy could be evaluated.

Complying with the results from other studies, which describe the three markers as characteristic of tumor cells, the results found here show that the studied markers were expressed in the analyzed tumor samples; blood samples collected at diagnosis had increased expression of the markers in relation to healthy women, opening the possibility of using these markers—especially *MCT1* and *CD147*—for diagnosis evaluations; in patients with disease progression the expression of these markers is even higher than in those without progression, so these markers—especially *MCT4* —could be used in prognosis evaluation. In comparison to MCF7 cells, the expression of the markers is low in healthy women, whereas in cancer patients this expression is higher, as expected; the obtained results, after validation, could be a promising strategy in routine laboratory evaluations as breast cancer diagnosis and prognosis. Considering that MCTs and *CD147* differential expression is found in several kinds of neoplasias and CTCs are tumor cells disseminated in peripheral blood, the markers studied here could also be useful in diagnosis and prognosis of cancers other than breast cancer, such as pancreas, kidney, stomach, lung, etc.

## Figures and Tables

**Table 1:** Clinical characterization of included patients.

Characteristics	*n*	%
**Clinical Stage**		
0/I	36	28.8
II	59	47.2
III	30	24.0
**Progression**		
Negative	113	91.9
Positive	10	8.1
**Hormone Receptors Expression**		
Negative	33	26.8
Positive	90	73.2
**Estrogen Receptor**		
Negative	31	25.2
Positive	92	74.8
**Progesterone Receptor**		
Negative	51	41.5
Positive	72	58.5
Her_ihc *		
Negative	28	22.9
+/3+	39	32.0
++/3+	24	19.7
+++/3+	31	25.4
	Median (p.25; p.75)	Min.; Max.
Age	52.5 (47; 61)	27; 78
Follow-up Time (months)	28 (24.5; 32)	4; 49

p.25; p.75: 25–75 percentiles respectively; Min.: Minimum; Max.: Maximum; * Immunohistochemistry Her2 tumor *status* classification according to good practices in pathology; 3+ = Her2 positive tumors.

**Table 2:** Difference in expression of monocarboxylate transporters (MCTs) and CD147 in tumors in relation to the clinical stage.

Gene	Difference in Expression (Fold Change)			
	Stage II versus 0/I	Stage III versus II	Stage III versus 0/I	*p* *
*MCT1*	0.65 (0.43–0.98)	27.46 (18.12–41.62)	17.78 (11.73–26.25)	0.149
*MCT4*	7.12 (4.7–10.8)	1.25 (0.67–2.32)	8.89 (5.86–13.47)	0.433
*CD147*	3.70 (3.23–4.26)	1.84 (1.60–2.11)	6.83 (5.95–7.85)	0.050

* Kruskal–Wallis.

**Table 3:** Expression differences of studied markers in the peripheral blood of patients with cancer at diagnosis in relation to healthy women.

Gene	Expression Difference (Fold Change)	*p* *
*MCT1*	10.54 (5.3–21.01)	<0.001
*MCT4*	4.07 (2.03–8.15)	0.066
*CD147*	11.8 (5.13–27.10)	<0.001

* Mann–Whitney; calibrator group: healthy women.

**Table 4:** Difference in expression of MCT and *CD147* markers from blood samples of patients at diagnosis, healthy women and MCF7 (calibrator).

Sample	Disease Status	*MCT1* *	*MCT4* *	*CD147* *
Donor	Healthy	0.02 (0.01–0.05)	0.001 (0.0005–0.002)	0.067 (0.063–0.072)
Patient	Without Progression	0.15 (0.08–0.28)	0.003 (0.001–0.006)	0.35 (0.32–0.37)
	With Progression	0.58 (0.31–1.09)	0.056 (0.03–0.08)	0.65 (0.53–0.80)

* In all analysis, *p* < 0.0001.
